# Increased B4GALT1 expression associates with adverse outcome in patients with non-metastatic clear cell renal cell carcinoma

**DOI:** 10.18632/oncotarget.8737

**Published:** 2016-04-15

**Authors:** Huyang Xie, Yu Zhu, Huimin An, Hongkai Wang, Yao Zhu, Hangcheng Fu, Zewei Wang, Qiang Fu, Jiejie Xu, Dingwei Ye

**Affiliations:** ^1^ Department of Urology, Fudan University Shanghai Cancer Center, Shanghai, China; ^2^ Department of Oncology, Shanghai Medical College, Fudan University, Shanghai, China; ^3^ Department of Biochemistry and Molecular Biology, School of Basic Medical Sciences, Fudan University, Shanghai, China

**Keywords:** clear cell renal cell carcinoma, B4GALT1, overall survival, prognostic biomarker, nomogram

## Abstract

*B4GALT1* is one of seven beta-1, 4-galactosyltransferase (B4GALT) genes, which has distinct functions in various malignances. Here, we evaluate the association of B4GALT1 expression with oncologic outcome in patients with non-metastatic clear cell renal cell carcinoma (ccRCC). A retrospective analysis of 438 patients with non-metastatic ccRCC at two academic medical centers between 2005 and 2009 was performed. The first cohort with 207 patients was treated as training cohort and the other as validation cohort. Tissue microarrays (TMAs) were created in triplicate from formalin-fixed, paraffin embedded specimens. Immunohistochemistry (IHC) was performed and the association of B4GALT1 expression with standard pathologic features and prognosis were evaluated. B4GALT1 expression was significantly associated with tumor T stage (*P*<0.001 and *P*<0.001, respectively), Fuhrman grade (*P*<0.001 and *P*<0.001, respectively) and necrosis (*P*=0.021 and *P*=0.002, respectively) in both training and validation cohorts. And high B4GALT1 expression indicated poor overall survival (OS) (*P*<0.001 and *P*<0.001, respectively) in the two cohorts. Furthermore, B4GALT1 expression was identified as an independent adverse prognostic factor for survival (*P*=0.007 and *P*=0.002, respectively). Moreover, the accuracy of established prognostic models was improved when B4GALT1 expression was added. Therefore, a predictive nomogram was generated with identified independent prognosticators to assess patients' OS at 5 and 10 years. Increased B4GALT1 expression is a potential independent adverse prognostic factor for OS in patients with non-metastatic ccRCC.

## INTRODUCTION

Clear cell renal cell carcinoma (ccRCC) is the most common subtype of kidney cancer and its incidence is increasing by 2.5% annually all over the world [1]. As reported in the latest cancer statistics, kidney cancer is one of the ten most common cancers in the US [2]. Although the diagnosis and treatment of ccRCC is evolving continuously, about 20% to 30% patients present with metastases at initial diagnosis. In addition, a third of patients after curable resection of the tumors progress to metastatic disease or experience local recurrence during long term follow-up [3]. However, metastatic ccRCC which is highly resistant to radiotherapy and chemotherapy, has a poor prognosis that 5-year survival rate is less than 10% [4]. Currently, several clinical and pathological factors, including TNM stage, Fuhrman nuclear grade, ECOG performance status (ECOG-PS) and tumor necrosis, are widely used for RCC prognosis. And some integrated prognostic models based on these factors has been established to predict patients' clinical outcome, such as University of California Integrated Staging System (UISS) and Mayo Clinic stage, size, grade and necrosis score (SSIGN) [5]. Since RCC is a complex, heterogeneous disease, the use of these predictors and integrated models are not always particularly accurate. Moreover, the clinical significance of biological markers is underestimated at most time. Combining them with the conventional clinicopathological predictors might improve current prognostic models.

The β4-galactosyltransferase (B4GALT) exists as a family that consists of seven members with distinct acceptor specificities, tissue distribution, chronological expression, and biological functions [6]. Following the action of B4GALTs, many other glycosyltransferases compete each other to modify the galactosylated glycans to express biologically active carbohydrate determinants such as poly-N-acetyllactosamine, polysialic acid, HNK-1 antigen, and Lewis X and sialyl-Lewis X determinants, to develop several series of glycolipids, and to extend chondroitin sulfate and heparin sulfate chains. Therefore, the β4-galactosylation of the glycans is quite important for many biological events including the development of cancers. Changes in the expression of the β4GalT genes have been reported in several types of cancers, such as lung cancer [7], liver cancer [8], breast cancer [9], leukemia [10], neuroblastoma [11], prostate cancer [12] and colon cancer [13, 14] and so on, correlated with cancer cell proliferation, metastasis, invasiveness and drug resistance.

Since our lab's former work on B4GALTs in lung cancer, liver cancer and glioma suggested its vital role in cancers, we analyzed B4GALT1 expression by IHC in ccRCC clinical specimens and its association with clinicopathological characteristics and clinical outcome. We further generated a nomogram with identified independent prognosticators and B4GALT1 to assess patients' OS rate at 5 and 10 years.

## RESULTS

### Immunohistochemical B4GALT1 intensity and its association with pathological characteristics

**Figure 1:**
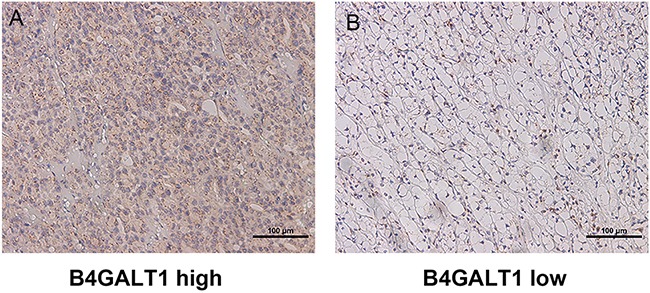
B4GALT1 expression in clear cell carcinoma (ccRCC) tissues Representative B4GALT1 immunohistochemistry (IHC) images of ccRCC tissue with high expression level **A.** and low expression **B.**

To investigate whether B4GALT1 expression is related to ccRCC development and progression, we first evaluated its expression by IHC in both training cohort and validation cohort. As presented in Figure 1, B4GALT1 positive staining was predominantly located in the cytoplasm and presented as dot-shaped stain with variable numbers. According to the cutoff value derived from IRS score by X-tile aforementioned, we separated the two cohorts into high (n=95 and n=109) and low (n=112 and n=122) B4GALT1 expression groups, respectively.

The detailed characteristics of patients and the correlation between B4GALT1 expression and clinicopathological features are listed in Table 1. There were 38 (18.4%) patients died in the training cohort due to all causes during the follow-up period, while 38 (16.5%) patients died in the validation cohort. The two cohorts were mostly matched for pathological characteristics as showed in Table 1. B4GALT1 expression was significantly associated with tumor T stage (*P* < 0.001 and *P* < 0.001, respectively; Table 1) and Fuhrman grade (*P*<0.001 and *P*<0.001, respectively; Table 1) and necrosis (*P*=0.021 and *P*=0.002, respectively; Table 1) in the training cohort and validation cohort.

**Table 1:** Association between B4GALT1 expression and patient characteristics

Characteristic	Training set (n=207)					Validation set (n=231)				
	Patients		B4GALT1 expression			Patients		B4GALT1 expression		
	NO.	%	Low	High	*P*	NO.	%	Low	High	*P*
Age, years					0.230					0.896
Mean±SD	53.3±12.8		53.3±12.9	54.5±13.8		55.2±11.6		55.2±11.6	55.4±11.6	
Range, median	20-83,53		21-78,51	20-83,55		22-81,56		22-80,56	29-81,55	
Gender					0.941					0.313
Male	140	67.6	76	64		172	74.5	87	85	
Female	67	32.4	36	31		59	25.5	35	24	
Tumor size, cm					<0.001					0.185
Mean±SD	4.5±2.6		3.8±1.6	5.3±3.2		4.5±2.5		4.3±2.3	4.7±2.7	
Range	1.0-20.0		1.0-9.5	1.5-20.0		0.5-14.0		0.8-14.0	0.5-14.0	
T stage					**<0.001**					**<0.001**
T1	165	79.7	105	60		150	64.9	91	59	
T2	16	7.7	2	14		23	10.0	14	9	
T3	19	9.2	5	14		53	22.9	16	37	
T4	7	3.4	0	7		5	2.2	1	4	
Fuhrman grade					**<0.001**					**<0.001**
1	34	16.4	24	10		47	20.3	45	2	
2	117	56.5	71	46		100	43.3	58	42	
3	49	23.7	16	33		53	22.9	18	35	
4	7	3.4	1	6		31	13.4	1	30	
Necrosis					**0.021**					**0.002**
absent	188	90.8	107	81		188	81.4	109	79	
present	19	9.2	5	14		43	18.6	13	30	
ECOG					0.010					0.152
0	171	82.6	100	71		190	82.3	105	85	
≥1	36	17.4	12	24		41	17.7	17	24	

### High expression of B4GALT1 is associated with OS and is an independent indicator of poor survival in patients with non-metastatic ccRCC

**Figure 2:**
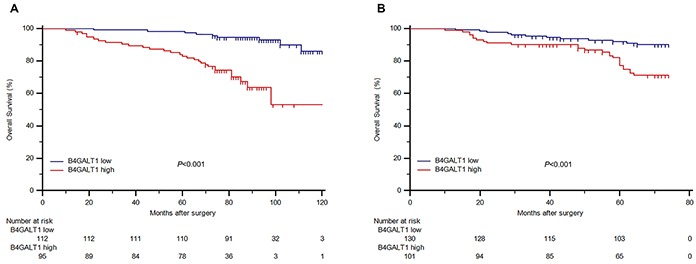
Overall survival (OS) analysis of patients with non-metastatic ccRCC based on B4GALT1 expression Kaplan-Meier analysis of OS in training cohort (n=207) **A.** and in validation cohort (n=231) **B.** *P* value was calculated by log-rank test.

Kaplan–Meier survival analysis was applied to compare OS according to the B4GALT1 expression. Patients with high B4GALT1 expression had a significantly poorer OS (*P* < 0.001 and *P* < 0.001, respectively; Figure 2A and 2B) than those with low B4GALT1 expression in both training and validation cohorts. To investigate whether this finding was independent of well-known prognostic indicators like tumor T stage, Fuhrman grade, necrosis and ECOG-PS, we performed univariate and multivariate Cox analyses of all the clinicopathological variables with B4GALT1 expression. As shown in Table 2, tumor T stage (HR = 6.535, CI: 3.320–12.864, *P* < 0.001 in training cohort; HR = 2.653, CI: 1.381–5.094, *P* = 0.004 in validation cohort), Fuhrman grade (HR = 3.899, CI: 2.060-7.380, *P* < 0.001 in training cohort; HR = 3.758, CI: 1.966–7.185, *P* < 0.001 in validation cohort), necrosis (HR = 5.348, CI: 2.584–11.067, *P* < 0.001 in training cohort; HR = 5.793, CI: 3.063–10.959, *P* < 0.001 in validation cohort), ECOG-PS (HR = 4.660, CI: 2.413–8.998, *P* < 0.001 in training cohort; HR = 4.551, CI: 2.332–8.879, *P* < 0.001 in validation cohort), and high expression of dichotomous B4GALT1(HR = 6.363, CI: 2.895–13.986, *P* < 0.001 in training cohort; HR = 3.082, CI: 1.560–6.090, *P* = 0.001 in validation cohort) were identified as risk factors, which might indicate poorer survival in patients with non-metastatic ccRCC.

**Table 2:** Univariate and multivariate Cox regression analysis of overall survival of the two cohorts

	Univariate			Multivariate		
	HR	95%CI	*P*	HR	95%CI	*P*
Training cohort						
B4GALT1	6.363	2.895-13.986	<0.001	3.234	1.388-7.536	0.007
ECOG	4.660	2.413-8.998	<0.001	3.075	1.555-6.082	0.001
Fuhrman	3.899	2.060-7.380	<0.001	2.207	1.105-4.408	0.026
Necrosis	5.348	2.584-11.067	<0.001	2.653	1.203-5.849	0.016
T stage	6.535	3.320-12.864	<0.001	3.473	1.692-7.127	<0.001
Validation cohort						
B4GALT1	3.082	1.560-6.090	<0.001	3.007	1.504-6.013	0.002
ECOG	4.551	2.333-8.879	<0.001	3.827	1.846-7.931	<0.001
Fuhrman	3.758	1.966-7.185	<0.001	2.914	1.436-5.914	0.003
Necrosis	5.793	3.063-10.959	<0.001	5.334	2.576-11.045	<0.001
T stage	2.653	1.381-5.094	0.004	4.661	2.201-9.868	<0.001

Then multivariate Cox regression analysis involving potential risk factors identified by univariate Cox analysis was performed. Besides tumor T stage, Fuhrman grade, and ECOG-PS, B4GALT1 expression was identified as an independent prognostic factor for OS (Table 2, dichotomous B4GALT1: HR = 3.234, CI: 1.388–7.536, *P* = 0.007 in training cohort; HR = 3.007, CI: 1.504–6.013, *P* = 0.002 in validation cohort).

### Extension of prognostic models with B4GALT1 expression for patients with non-metastatic ccRCC

To further assess the prognostic power of B4GALT1 expression, we constructed prognostic models combining B4GALT1 expression with SSIGN score and UISS score to compare the prognostic accuracy of these models by C-index and AIC analysis. As presented in Table 3, in the training cohort, the C-indices were 0.776 and 0.802 when assessed with SSIGN and UISS outcome algorithms and were improved to 0.803 and 0.819 when dichotomous B4GALT1 signature was added. Likewise, in the validation cohort, the C-indices were improved from 0.792 and 0.729 to 0.813 and 0.776 when dichotomous B4GALT1 signature was added. Furthermore, each combined model showed a lower AIC value than corresponding conventional model alone.

**Table 3:** Comparison of the accuracy of the prognostic models and B4GALT1 expression for overall survival

Model	Training cohort		Validation cohort	
	C-index	AIC	C-index	AIC
B4GALT1 (high vs. low)	0.702	358.585	0.688	381.961
UISS	0.802	334.279	0.729	374.231
UISS+B4GALT1	0.819	330.829	0.776	365.492
SSIGN	0.776	341.424	0.792	344.436
SSIGN+B4GALT1	0.803	338.847	0.813	337.208

### Prognostic nomogram of non-metastatic ccRCC

**Figure 3:**
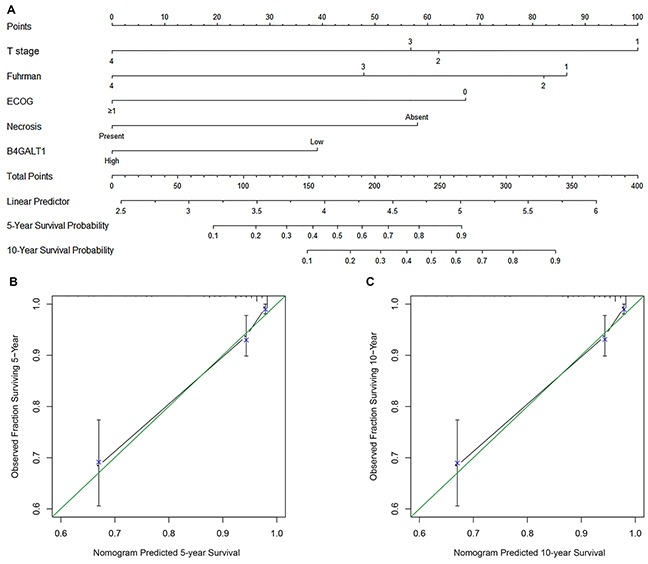
Nomogram and calibration plots for the prediction of overall survival (OS) in patients with non-metastatic ccRCC Nomogram to predict OS at 5 and 10 years after surgery **A.** the calibration plots for predicting OS at 5 years **B.** and 10 year **C.**

Based on the results arising from multivariate analysis of OS and along with the results of C-index and AIC analyses, we used the patients' data in the two cohorts to develop a nomogram to predict OS at 5 and 10 years after surgery (Figure 3A). The predictors included tumor T stage, ECOG-PS, Fuhrman grade, necrosis and B4GALT1 expression, all of which were independent prognostic indicators for OS. The calibration plots of the nomogram are shown for 5- and 10-year predictions (Figure 3B and 3C).

## DISCUSSION

To our knowledge, this study is the first to report an association between high expression of B4GALT1 and an increased risk of death in patients with non-metastatic ccRCC after surgery. High B4GALT1 expression was positively associated with histologic necrosis status, tumor T stage and Fuhrman grade, which strong indicated that B4GALT1 plays a critical role in ccRCC development and progression. What's more, the accuracy of SSIGN and UISS prognostic models was improved both when B4GALT1 expression was added.

It is well known that N-linked oligosaccharides on glycoprotein are structurally altered during malignant transformation. β1, 4-Galactosyltransferase (B4GALT) family is a class of enzymes responsible for the biosynthesis of N-acetyllactosamine on N-glycans by transferring UDP-galactose and consists of seven members, from B4GALT1 to B4GALT7. B4GALT5 and B4GALT6 also play a role in lactosylceramide synthesis [15]. B4GALT family is involved in complicated protein-protein interaction networks, which link glycosylation to other cellular processes. In cancer cells, the expression of B4GALT is regulated by many factors, including transcriptional factors and also extracellular factors. Transcriptional regulation is the most critical way in B4GALT regulation. As former study in our lab reported, B4GALT1 and B4GALT5 gene are both regulated by Ets1 transcription factor [7] and is involved in the regulation by EGF/RAS/ERK pathway of the two genes [16]. Exogenous reagents like etoposide could regulate B4GALT5 in glioma by influencing the level of related transcription factors [17].

The increased expression of B4GALT1 led to the formation of the sialyl-Lewis X determinant, which correlated with metastatic potentials of human lung cancer cells and U937 cells [10]. But in our study, the expression of B4GALT1 have not been correlated with patients' recurrence-free survival in multivariable analysis. B4GALT1 and B4GALT5 have been shown to be involved in the development of multidrug resistance of human leukemic cells by activating the hedgehog signaling and the expression of the transporters, p-glycoprotein, and multidrug resistance-associated protein 1 [6, 10]. Overexpression of B4GALT1 or B4GALT5 resulted in the acquirement of multidrug resistance in HL60 cells, and silencing of B4GALT1 or B4GALT5 resulted in the cells sensitive to therapeutic drugs [10]. Overexpression of B4GALT2 has been shown to induce p53-mediated apoptosis in HeLa cells [18]. The expression of B4GALT3 enhanced migration and invasion of human neuroblastoma cells [11], and highly expression of B4GALT4 was associated with colorectal cancer metastasis and poor prognosis [19]. In the cases of B4GALT6 and B4GALT7, no knowledge is available for their relevance to cancers.

Although the clinical significance of B4GALT1 expression in patients with non-metastatic ccRCC has been revealed in our study, some limitations remain to be resolved. Considering that all specimens from were collected from two tertiary referral hospitals, the results need to be further validated or revised in larger data sets and external heterogeneous cohorts. Although duplicate 1.0 or 1.5 mm tissue cores from three different areas were used to construct the TMA, the deviation of TMA analysis could not be avoided owing to the heterogeneous natural history of ccRCC. Moreover, the molecular role of B4GALT1 in ccRCC remains to be fully elucidated in our future study.

In summary, our present study identified B4GALT1 expression as a potential independent unfavorable prognostic indicator for OS of patients with non-metastatic ccRCC. Combining B4GALT1 expression with conventional prognostic models could improve their prognostic accuracy. Further researches might attempt to verify whether B4GALT1 could be developed as a new therapeutic target.

## MATERIALS AND METHODS

### Patients and clinical database

We performed a retrospective study in which enrolled 438 patients with non-metastatic ccRCC undergoing RN or NSS at two academic medical centers between 2005 and 2009. The 207 patients from Fudan University Shanghai Cancer Center represented our training cohort, and the other 238 patients from Zhongshan Hospital of Fudan University, Shanghai represented our validation cohort. All patients had paraffin-embedded tissue blocks available for IHC staining and outcome data. Patients were selected based on the following criteria, including 1) confirmed histopathology diagnosis, 2) no adjuvant anticancer therapy after surgery, and 3) reviewed TNM classification according to the 2010 American Joint Committee on Cancer. Clinicopathological information of each patient was obtained from patients' in-patient records. The Mayo Clinic Stage, Size, Grade, and Necrosis score (SSIGN) and University of Los Angeles Integrated Staging System (UISS) scores were used for the patients. Overall survival (OS) was the main end point of this study. OS was calculated from the date of surgery to the date of death or to the date of the latest follow-up. Median follow-up was 86 months (range: 10–127 months) in training cohort and 71 months (range: 10–74 months) in validation cohort. Ethical approval was granted both by the two hospitals' research medical ethics committees and informed consent was provided by all the patients enrolled in this study.

### Immunohistochemistry

Tissue microarrays were constructed as described previously [20]. Primary anti- B4GALT1 antibody (diluted 1:100; HPA010806; Sigma-Aldrich) was used for IHC staining. Specimen staining intensity was evaluated by two independent pathologists blinded to clinicopathological data and clinical outcome of each patient. A semi-quantitative immunoreactivity scoring (IRS) system was used for this evaluation as reported elsewhere [21, 22]. We respectively selected the optimum cutoff scores (110 and 116) for the staining intensity to separate patients of the two cohorts into high and low B4GALT1 expression groups by using X-tile software, version 3.6.1 (Yale University, New Haven, Connecticut).

### Statistical analysis

MedCalc and Stata 12.0 were used for statistical analysis. Categorical data were analyzed using the Fisher exact or chi-square test. Numerical data were analyzed by the Student t-test. Subgroup OS curves were calculated by the Kaplan-Meier method and compared by log rank test. We used univariate and multivariate Cox proportional hazard models to evaluate the HR and 95% CI. The accuracy of the prognostic factors were evaluated by Harrell's concordance index (C-index). Furthermore, the Akaike information criterion (AIC) value was calculated to evaluate the discriminatory ability of prognostic models, and smaller AIC values present a better predicting ability. We applied the R software and the “rms” package (R Foundation for Statistical Computing, Vienna, Austria) to perform the nomogram analysis and calibration plot. All statistical tests were two sided and *P*<0.05 was considered statistically significant.
